# CauSTI: causal spatial transcriptomic interaction inference for cell-cell communication

**DOI:** 10.3389/fcell.2026.1943696

**Published:** 2026-09-23

**Authors:** Hongjie Ji, Yue Huang, Yu Lyu

**Affiliations:** 1 Department of Information Security Technology, Jilin Police College, Changchun, China; 2 The First Clinical Medical College, Guangzhou University of Chinese Medicine, Guangzhou, China; 3 Faculty of Innovation Engineering, Macau University of Science and Technology, Taipa, Macao, China

**Keywords:** bioinformatics, cell-cell communication, graph neural networks, ligand-receptor inference, spatial transcriptomics

## Abstract

**Introduction:**

Cell-cell communication plays a critical role in tissue organization, development, immune regulation, and disease progression. Spatial transcriptomics enables the inference of such communication within the native tissue context. However, existing methods often rely on ligand–receptor co-expression or spatial proximity alone, without explicitly modeling the directional nature of intercellular signaling.

**Methods:**

We propose CauSTI, a causal spatial transcriptomic interaction inference framework for identifying direction-specific cell-cell communication. CauSTI integrates spatial neighborhood structure with ligand–receptor-derived causal priors through a causal-prior-guided variational graph autoencoder. Spatial proximity defines the tissue graph, while ligand–receptor evidence provides biologically informed directional constraints. An asymmetric decoder models sender–receiver relationships between ordered cell pairs. The optimization objective jointly incorporates spatial reconstruction, causal-prior consistency, variational regularization, and directional regularization.

**Results:**

Experiments on three spatial transcriptomic datasets—seqFISH+, MERSCOPE, and Xenium—demonstrate that CauSTI consistently outperforms representative baseline methods in AUROC, ACC, and AP. Ablation studies further confirm the contributions of causal priors and directional regularization.

**Discussion:**

These findings suggest that integrating spatial context with causal priors and explicit directional modeling provides an effective framework for inferring biologically plausible, direction-specific cell-cell communication landscapes from spatial transcriptomic data.

## 1 Introduction

Cell-cell communication (CCC) plays a fundamental role in tissue organization, immune regulation, development, and disease progression Su et al. (2024). Through ligand-receptor interactions, cells exchange biochemical signals with neighboring or distant cells, thereby coordinating cellular states and collective tissue functions Yang et al. (2021). With the rapid development of single-cell RNA sequencing and spatial transcriptomics, it has become possible to investigate CCC not only from gene expression profiles but also within the native spatial context of tissues Gulati et al. (2025). Compared with dissociated single-cell data, spatial transcriptomics provides positional information for each spot or cell, offering a unique opportunity to identify communication events that are both molecularly plausible and spatially constrained.

A number of computational methods have been developed to infer CCC from transcriptomic data Wilk et al. (2024). Classical methods such as CellPhoneDB Efremova et al. (2020), CellChat Jin et al. (2021), and NicheNet Browaeys et al. (2020) mainly rely on curated ligand-receptor databases and evaluate communication probability between predefined cell types or cell groups. These approaches have greatly advanced the systematic analysis of intercellular signaling, but they are often limited by the loss of spatial context or by group-level inference that may overlook cell-level heterogeneity Xiao et al. (2026). More recent spatially aware methods, such as stLearn Pham et al. (2023) and SpaTalk Shao et al. (2022), have incorporated spatial proximity, ligand-receptor co-expression, downstream signaling, or optimal transport frameworks to improve CCC inference in spatial transcriptomics. Some large language models for CCC have further expanded the scope of CCC by integrating richer biological backgrounds and more flexible learning paradigms Ji et al. (2024), Ji et al. (2026). These methods demonstrate that integrating molecular interactions with spatial organization is essential for reliable identification of tissue-specific communication patterns Wang et al. (2025).

Despite these advances, several challenges remain. First, many existing methods rely primarily on ligand-receptor co-expression or spatial colocalization scores, which may not fully capture the latent cellular state and higher-order neighborhood context encoded in spatial tissue graphs. Second, although CCC is inherently directional, with ligand-expressing sender cells acting on receptor-expressing receiver cells, many graph-based representation learning methods are designed for undirected spatial graphs and do not explicitly model asymmetric sender-receiver relationships. Third, current approaches often treat spatial neighborhood information and ligand-receptor prior knowledge as separate scoring or post-processing components, rather than integrating them into an end-to-end trainable framework with explicit regularization toward biologically informed directional priors.

To address these limitations, we propose CauSTI, a Causal Spatial Transcriptomic Interaction network inference framework for direction-specific CCC prediction. CauSTI integrates spatial neighborhood structure with ligand-receptor-derived causal priors in a variational graph autoencoder. Specifically, spatial proximity is used to construct a tissue graph, while ligand-receptor evidence provides directional prior information from sender cells to receiver cells. CauSTI then learns latent cell representations through a causal-prior-guided graph encoder and predicts ordered cell-cell interaction probabilities using an asymmetric decoder. A spatial-causal prior-guided optimization objective further combines spatial graph reconstruction, causal-prior consistency, variational regularization, and directional regularization.

The main contributions of this study are summarized as follows:

- We develop a ligand-receptor-informed variational graph autoencoder that jointly encodes spatial neighborhood structure and gene expression profiles for cell-level communication inference.
- We design a multi-objective regularization strategy that integrates spatial graph reconstruction, ligand-receptor prior consistency, variational regularization, and anti-symmetric constraints, thereby improving both biological interpretability and directional specificity of inferred communications.
- Experiments on seqFISH+, MERSCOPE, and Xenium datasets demonstrate that CauSTI outperforms representative baseline methods, and ablation studies confirm the importance of both causal priors and directional regularization.

## 2 Related work

The main methods for inferring intercellular communication using spatial data can be broadly classified into two categories: methods based on optimal transport theory and methods based on graph neural networks. Optimal transport-based methods model spatial transcriptomic data as distributions and infer correspondence or signal flow by minimizing transport costs under spatial and molecular constraints. Early OT-based frameworks such as novoSpaRc Moriel et al. (2021) reconstruct spatial gene-expression maps by formulating single-cell spatial assignment as a generalized optimal transport problem. SpaOTsc Cang and Nie (2020) further extends this idea to cell-cell communication inference by first recovering a spatial metric for single cells and then estimating ligand-receptor-mediated signaling through optimal transport between signal senders and receivers. More recently, COMMOT Cang et al. (2023) proposed a collective optimal transport framework to infer spatially resolved communication by jointly considering multiple ligand-receptor pairs, molecular competition, and spatial distance constraints. In addition, PASTE Liu et al. (2023) applies optimal transport to align and integrate adjacent spatial transcriptomic slices, providing improved spatial registration and tissue reconstruction that can support downstream communication analysis. These OT-based methods are attractive because they provide mathematically interpretable transport plans and naturally incorporate spatial distance. However, they often depend on the definition of transport costs, the quality of spatial alignment, and prior ligand-receptor annotations.

Graph neural network-based methods, in contrast, represent spatial transcriptomic data as graphs, where nodes correspond to cells or spots and edges encode spatial proximity, histological similarity, or potential biological interactions Huo and Zhang (2026). DeepLinc Li and Yang (2022) formulates the reconstruction of proximal and distal cell-cell interactions as a graph representation learning problem and employs a variational graph autoencoder-based generative framework to estimate missing interactions. scDCA Ji et al. (2025) uses a multi-view graph convolutional network to reconstruct CCC maps at single-cell resolution, and then uses an attention mechanism to interpret the model to explore cell communication mechanisms. GCNLA Yang et al. (2025) further combines graph convolutional networks with an LSTM-based attention mechanism, allowing the model to capture both local spatial dependencies and structural relationships among cells for cell-cell interaction inference. OrgaCCC Feng et al. (2025) introduces a dual-branch orthogonally coupled variational graph autoencoder to jointly learn representations from cellular and gene-level graphs. By aligning reconstructed features across these two hierarchical levels, OrgaCCC enables cross-level information fusion and improves the accuracy of cell-cell communication inference.

## 3 Methods

We developed a causal-prior-regularized variational graph autoencoder, termed CauSTI, to infer directed cell-cell interaction landscapes from spatial transcriptomic data (Figure 1). The model extends a graph autoencoder framework by jointly using spatial proximity and ligand-receptor-derived causal priors. Spatial proximity defines putative physical contact between cells, whereas ligand-receptor evidence provides directional biological constraints from sender cells to receiver cells. The model learns latent cell representations from gene expression and spatial adjacency, then decodes directed interaction probabilities between all ordered cell pairs.

**FIGURE 1:**
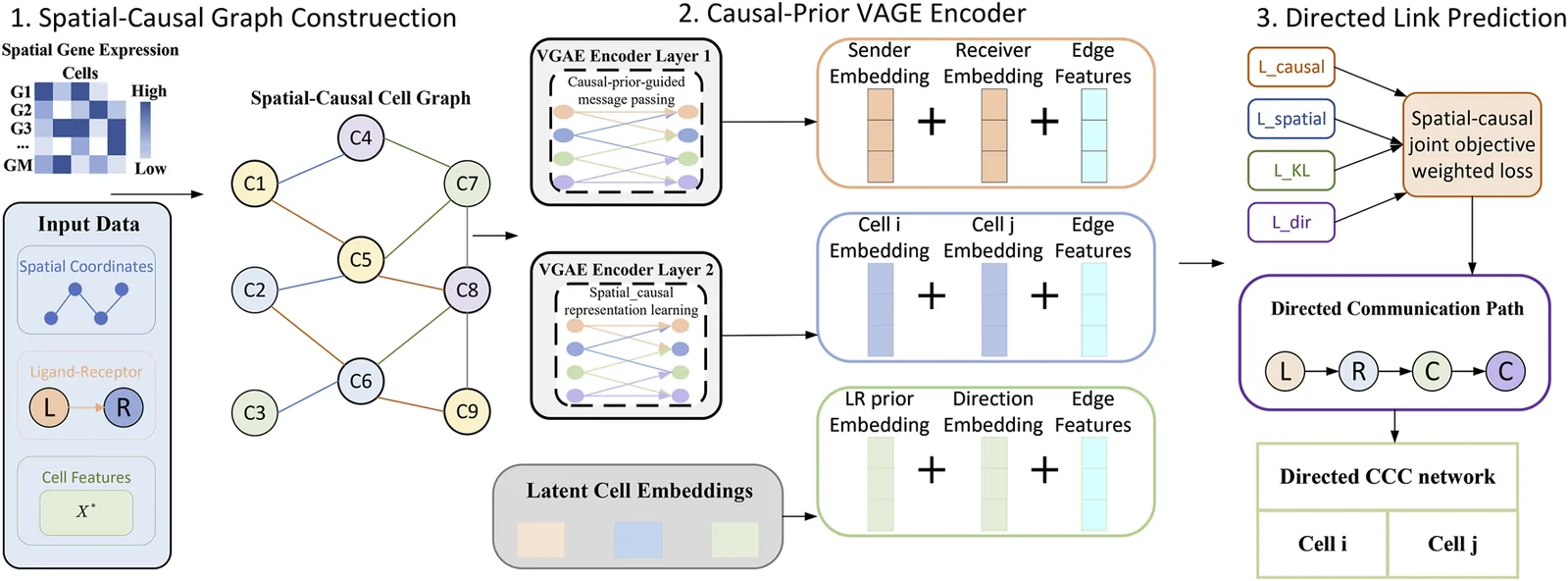
CauSTI infers direction-specific cell-cell communication from spatial transcriptomic data through three main stages. First, spatial gene expression profiles, cell coordinates, ligand-receptor prior knowledge, and cell-level features are integrated to construct a spatial-causal cell graph, where spatial neighborhood edges encode local proximity and ligand-receptor edges provide directional causal priors. Second, a causal-prior-guided variational graph autoencoder learns latent cell embeddings through spatial- and causal-aware message passing. Finally, multiple objectives, including causal-prior consistency, spatial reconstruction, variational regularization, and directional regularization, are jointly optimized to generate a directed sender-to-receiver cell-cell communication network.

### 3.1 Collection of L-R pairs

For the analysis of real-world datasets, we curated ligand-receptor interaction information from five publicly available resources, including the International Union of Pharmacology (Sharman et al., 2012), CellTalkDB (Shao et al., 2021), Human Plasma Membrane Receptome (Ben-Shlomo et al., 2003), HPRD (Prasad et al., 2009), Fantom5 (Ramilowski et al., 2015). After integration, the resulting comprehensive ligand-receptor reference contained 2,479 mouse and 3,057 human L-R pairs.

### 3.2 Spatial-causal graph construction

Let $X^{\mathrm{raw}}\in R^{n\times p}$ denote the raw gene expression matrix, where n is the number of spatial spots or cells and p is the number of genes. Each cell i is associated with a spatial coordinate $s_{i}=\left(x_{i},y_{i}\right)$ . The raw count matrix is first subjected to library size normalization and logarithmic transformation in Equation 1:

$$X_{ig}=\log\left(1+\frac{X_{ig}^{\mathrm{raw}}}{\overset{p}{\underset{g^{\prime }=1}{\sum }}X_{ig^{\prime }}^{\mathrm{raw}}}\alpha \right)$$ (1)

where α is a scaling factor. Highly variable genes are then selected to obtain the processed expression matrix $X^{*}\in R^{n\times d}$ .

Spatial proximity is modeled using a k -nearest-neighbor graph. In Equation 2, the Euclidean distance between cells i and j is defined as

$$d_{ij}=\|s_{i}-s_{j}{\|}_{2}$$ (2)

In Equation 3 the spatial adjacency matrix is given by

$$A_{ij}^{sp}=\left\{\begin{array}{cc} 1, & j\in N_{k}\left(i\right),\;i\neq j,\\ 0, & \text{otherwise}. \end{array}\right.$$ (3)

Ligand-receptor information is incorporated as a directed causal prior. Given a ligand-receptor set P={(l,r)} , In Equation 4 the causal-prior score from source cell i to target cell j is computed as

$$C_{ij}=\underset{\left(l,r\right)\in P}{\sum }X_{il}^{*}X_{jr}^{*}K\left(d_{ij}\right)$$ (4)

where $X_{il}^{*}$ and $X_{jr}^{*}$ denote the expression levels of ligand l in cell i and receptor r in cell j , respectively. In Equation 5 the spatial decay kernel is

$$K\left(d_{ij}\right)=\exp\left(-\frac{d_{ij}}{\tau }\right)$$ (5)

where τ controls the spatial communication range. This causal-prior score is intended to provide a biologically informed but simplified directional constraint. It captures ligand availability in the sender cell, receptor availability in the receiver cell, and spatial attenuation between the 2 cells, but it does not explicitly model receptor-specific binding affinity, ligand competition, downstream signaling cascades, or receiver-cell-type-specific intracellular context. Thus, the prior should be interpreted as evidence for plausible sender-receiver communication rather than as a complete mechanistic representation of signal transduction.

In Equation 6, strong causal-prior edges are selected from the upper quantile of nonzero causal-prior scores:

$$A_{ij}^{ca}=\left\{\begin{array}{cc} 1, & C_{ij}\geq q_{\rho }\left(\left\{C_{uv}:C_{uv}>0,\;u\neq v\right\}\right),\\ 0, & \text{otherwise}. \end{array}\right.$$ (6)

In Equation 7, the hybrid adjacency matrix is then constructed as

$$\tilde{A}=A^{sp}+\lambda_{c}A^{ca}$$ (7)

and normalized in Equation 8:

$$\hat{A}={\tilde{D}}^{-1/2}\left(\tilde{A}+I\right){\tilde{D}}^{-1/2}$$ (8)

where $\tilde{D}$ is the degree matrix of $\tilde{A}+I$ .

### 3.3 Causal-prior VGAE encoder

The encoder learns latent representations from $X^{*}$ and $\hat{A}$ . In contrast to using the ligand-receptor score alone, this representation learning step allows CauSTI to incorporate broader transcriptional programs and local spatial context when estimating interaction probabilities. In Equation 9 a graph convolutional layer is defined as

$$GCN\left(X^{*},\hat{A}\right)=\sigma \left(\hat{A}X^{*}W\right)$$ (9)

where W is a trainable weight matrix and σ(⋅) is a nonlinear activation function.

In Equation 10 the hidden representation is computed as

$$H=GCN\left(X^{*},\hat{A}\right)$$ (10)

Two graph convolutional branches estimate the mean and log- variance of the latent distribution in Equations 11, 12

$$\mu ={GCN}_{\mu }\left(H,\hat{A}\right)$$ (11)

$$\log\sigma^{2}={GCN}_{\sigma }\left(H,\hat{A}\right)$$ (12)

In Equation 13 the latent embedding of cell iis sampled using the reparameterization trick:

$$z_{i}=\mu_{i}+\sigma_{i}⊙\epsilon_{i},\;\epsilon_{i}∼N\left(0,I\right)$$ (13)

In Equation 14 the approximate posterior is therefore

$$q\left(Z|X^{*},\hat{A}\right)=\overset{n}{\underset{i=1}{\prod }}N\left(z_{i}|\mu_{i},diag\left(\sigma_{i}^{2}\right)\right)$$ (14)

### 3.4 Directed link prediction

For each ordered cell pair (i,j) , the model predicts a directed interaction score using the source embedding $z_{i}$ , target embedding $z_{j}$ , and edge-level features $e_{ij}$ . In Equation 15 the asymmetric decoder is defined as

$$s_{ij}=z_{i}^{⊤}W_{d}z_{j}+u^{⊤}\left[z_{i}\|z_{j}\|e_{ij}\right]+b$$ (15)

where $W_{d}$ is an asymmetric bilinear matrix, u and b are trainable parameters, and ‖ denotes vector concatenation. In Equation 16 the directed interaction probability is

$${\hat{P}}_{ij}=\sigma \left(s_{ij}\right)$$ (16)

Since **W_d_** is not constrained to be symmetric. In Equation 17 the model can produce direction-specific predictions:

$${\hat{P}}_{ij}\neq {\hat{P}}_{ji}$$ (17)

### 3.5 Spatial-causal prior-guided optimization

The model is optimized by jointly leveraging spatial proximity and ligand-receptor-derived causal priors. The optimization process uses two complementary sources of prior knowledge: the spatial neighborhood structure encoded by $A^{sp}$ and the directed causal-prior graph encoded by $A^{ca}$ . Spatially adjacent cell pairs provide structural constraints for reconstructing local tissue organization, whereas ligand-receptor-supported ordered pairs provide biological constraints for inferring direction-specific communication.

In Equation 18, the spatial reconstruction objective encourages the predicted interaction probabilities to preserve local spatial organization:

$$\begin{array}{cc} L_{sp} & =-\underset{i\neq j}{\sum }\left[A_{ij}^{sp}\log{\hat{P}}_{ij}+\left(1-A_{ij}^{sp}\right)\log\left(1-{\hat{P}}_{ij}\right)\right] \end{array}$$ (18)

In Equation 19 the causal-prior consistency objective encourages the model to assign high probabilities to ordered cell pairs supported by strong ligand-receptor evidence:

$$\begin{array}{cc} L_{ca} & =-\underset{i\neq j}{\sum }\left[A_{ij}^{ca}\log{\hat{P}}_{ij}+\left(1-A_{ij}^{ca}\right)\log\left(1-{\hat{P}}_{ij}\right)\right] \end{array}$$ (19)

To regularize the latent representation space, in Equation 20 the approximate posterior is constrained toward a standard Gaussian prior through the Kullback-Leibler divergence:

$$L_{KL}=-\frac{1}{2}\overset{n}{\underset{i=1}{\sum }}\overset{h}{\underset{m=1}{\sum }}\left(1+\log\sigma_{im}^{2}-\mu_{im}^{2}-\sigma_{im}^{2}\right)$$ (20)

Because cell-cell communication is direction-dependent, In Equation 21 a directional regularization term is further introduced to reduce unsupported bidirectional predictions:

$$L_{\mathrm{dir}}=\underset{i<j}{\sum }{\hat{P}}_{ij}{\hat{P}}_{ji}$$ (21)

In Equation 22, the final optimization objective integrates the four terms:

$$L=\lambda_{sp}L_{sp}+\lambda_{ca}L_{ca}+\lambda_{KL}L_{KL}+\lambda_{\mathrm{dir}}L_{\mathrm{dir}}$$ (22)

where $\lambda_{sp}$ , $\lambda_{ca}$ , $\lambda_{KL}$ , and $\lambda_{\mathrm{dir}}$ are hyperparameters controlling the relative contributions of spatial reconstruction, causal-prior consistency, latent regularization, and directional regularization, respectively.

## 4 Experiment

### 4.1 Dataset

We conducted experiments on three spatial transcriptomics datasets generated using diverse sequencing- and imaging-based technologies, including seqFISH+ Eng et al. (2019), MERSCOPE Colbert et al. (2023) and Xenium Umek et al. (2025). These datasets cover different spatial resolutions, transcriptomic feature dimensions, and cellular composition, thereby providing a comprehensive benchmark for evaluating the proposed method under heterogeneous spatial transcriptomic settings.

SeqFISH+: The seqFISH + dataset was generated using sequential fluorescence *in situ* hybridization, a highly multiplexed imaging-based spatial transcriptomics technology. This dataset contains 913 spatially resolved cells, 10,000 measured gene features, and nine annotated node types.

MERSCOPE: The MERSCOPE dataset was obtained using a multiplexed error-robust fluorescence *in situ* hybridization platform. It includes 71,381 spatial nodes, 550 measured features, and 12 node types. Compared with transcriptome-wide sequencing-based datasets, MERSCOPE provides targeted but highly resolved spatial measurements.

Xenium: The Xenium dataset was profiled using the 10x Genomics Xenium platform, an imaging-based single-cell spatial transcriptomics technology. The dataset comprises 162,628 spatial nodes, 17,943 gene-expression features, and nine node types.

### 4.2 Evaluation of the model

To ensure a fair comparison with existing spatial cell-cell interaction inference methods, all methods were evaluated using the same spatial-neighborhood-based reference labels. Since most competing methods define potential interacting cell pairs according to spatial proximity, we used the spatial adjacency matrix as the common evaluation reference. This design allows different methods to be compared under a unified criterion, regardless of whether they explicitly incorporate ligand-receptor priors, causal priors, or graph representation learning.

Specifically, for each ordered non-self cell pair (i,j) , the binary reference label was defined as $y_{ij}=A_{ij}^{sp}$ , where $y_{ij}=1$ denotes a spatially adjacent candidate interaction and $y_{ij}=0$ denotes a non-adjacent pair. Each method produced a continuous interaction score or probability ${\hat{P}}_{ij}$ for the same set of candidate pairs. AUROC was calculated by ranking cell pairs according to ${\hat{P}}_{ij}$ and sweeping the decision threshold over all possible score values to obtain the receiver operating characteristic curve. For a threshold θ , a pair was predicted as positive if ${\hat{P}}_{ij}\geq \theta$ and negative otherwise. True positives (TP) were pairs with $y_{ij}=1$ and ${\hat{P}}_{ij}\geq \theta$ ; false positives (FP) were pairs with $y_{ij}=0$ and ${\hat{P}}_{ij}\geq \theta$ ; true negatives (TN) were pairs with $y_{ij}=0$ and ${\hat{P}}_{ij}<\theta$ ; and false negatives (FN) were pairs with $y_{ij}=1$ and ${\hat{P}}_{ij}<\theta$ . The true-positive rate and false-positive rate were computed as TPR=TP/(TP+FN) and FPR=FP/(FP+TN) , respectively, and AUROC was obtained as the area under the TPR-FPR curve. ACC was calculated as (TP+TN)/(TP+FP+TN+FN) using a probability threshold of 0.5. AP was calculated from the precision-recall curve using the same continuous scores and was used to evaluate ranking performance under class imbalance, since spatially adjacent cell pairs usually account for only a small fraction of all possible cell pairs.

We note that these spatial-neighborhood-based labels should be interpreted as proxy labels for benchmarking rather than direct experimental ground truth for signaling. Spatial adjacency enables reproducible and comparable evaluation across datasets, but it does not by itself prove that a ligand-receptor interaction is active. Conversely, some signaling events may occur between cells that are not immediate nearest neighbors. Therefore, the quantitative results reported here mainly evaluate whether each method can recover spatially constrained candidate interactions under a common benchmark. Independent biological validation, such as enrichment of known ligand-target gene effects in receiver cells or perturbation-based confirmation of predicted sender-receiver relationships, would provide stronger evidence for the biological activity of the inferred interactions when such data are available.

Because the spatial adjacency matrix is not direction-specific, the AUROC, ACC, and AP values in the main benchmark should not be interpreted as direct measurements of sender-to-receiver direction prediction. These metrics evaluate whether a method recovers spatially constrained candidate cell pairs. A fully direction-specific benchmark would require independent ordered-pair labels, for example, labels indicating that cell i signals to cell j but not necessarily from j to i , derived from perturbation experiments, ligand-target response evidence, or other validated downstream activity measurements. In the absence of such directed ground-truth labels for the evaluated datasets, the directional component of CauSTI is assessed through the model design, the directed ligand-receptor prior, the asymmetric decoder, and the ablation study removing the direction loss. Thus, the current experiments support the usefulness of direction-aware modeling indirectly, whereas independent direction-specific validation remains an important future evaluation.

### 4.3 Experimental setup details

All experiments were conducted on the same local workstation to ensure a consistent computational environment across methods. The workstation was equipped with a 13th Gen Intel(R) Core(TM) i9-13980HX processor with 32 logical threads, 31.63 GB RAM, and an NVIDIA GeForce RTX 4070 Laptop GPU with 8,188 MiB GPU memory. The GPU driver version was 546.80, with CUDA 12.3 supported by the installed NVIDIA driver. The operating system was 64-bit Windows 11.

The experimental environment was based on Python 3.11.5 from the Anaconda distribution. The spatial neighborhood graph was constructed using k=5 nearest neighbors. Gene expression profiles were first log-normalized, and the top 3,000 highly variable genes were selected as model input features. When a dataset contained fewer than 3,000 measured features, all available features were retained after normalization. The hidden dimension of the graph encoder was set to 128, and the latent embedding dimension was set to 64. The model was trained for 100 epochs using the Adam optimizer with a learning rate of $4\times 10^{-4}$ and a weight decay of $1\times 10^{-5}$ . For each dataset and each method, experiments were repeated 50 times with different random initializations.

### 4.4 Parameter tuning

CauSTI contains several parameters that control the spatial scale and the relative contribution of each optimization term. In our experiments, we used the same preprocessing strategy and a fixed k=5 nearest-neighbor graph to provide a consistent benchmark across datasets. When applying CauSTI to new spatial transcriptomic datasets, these parameters should be interpreted in relation to the spatial resolution, cell density, and expected communication range of the tissue.

The neighborhood size k controls the locality of the spatial graph. For high-resolution single-cell platforms with dense cell sampling, a small value such as k=3 to five is generally appropriate because it emphasizes immediate cellular neighborhoods. For sparser spot-level data or tissues with larger expected communication neighborhoods, larger values such as k=8 to 15 may be considered. In practice, k should be chosen so that the graph captures local tissue organization without connecting biologically distant regions.

The decay parameter τ controls how rapidly ligand-receptor evidence decreases with spatial distance. A practical strategy is to set τ according to the distribution of distances among spatial-neighborhood edges, for example, using the median or upper quartile of k -nearest-neighbor distances as an initial value. Smaller τ values emphasize short-range communication, whereas larger values allow more spatially extended paracrine effects. The causal-prior quantile threshold ρ similarly controls the sparsity of the directed ligand-receptor prior graph: higher thresholds retain only the strongest ligand-receptor-supported edges, while lower thresholds increase sensitivity at the cost of adding weaker candidate interactions.

For the loss weights $\lambda_{sp}$ , $\lambda_{ca}$ , $\lambda_{KL}$ , and $\lambda_{\mathrm{dir}}$ , we recommend first normalizing the reconstruction and causal-prior losses by the number of evaluated ordered cell pairs and then selecting weights so that no single term dominates the early training objective. The spatial and causal-prior terms should be balanced according to the intended use: increasing $\lambda_{sp}$ emphasizes recovery of local tissue structure, whereas increasing $\lambda_{ca}$ gives stronger influence to ligand-receptor-supported directionality. The KL term should be monitored to avoid overly diffuse or collapsed latent embeddings, and $\lambda_{\mathrm{dir}}$ should be tuned conservatively because some biological interactions may be genuinely reciprocal. When independent validation data are unavailable, parameter choices can be assessed by prediction stability across random seeds and by the enrichment of top-ranked interactions for known ligand-receptor pairs or plausible cell-type-specific communication patterns.

### 4.5 Comparison with other methods

We evaluated the proposed CauSTI model on three real-world spatial transcriptomic datasets, including seqFISH+, MERSCOPE, and Xenium. The performance was compared with three representative baseline methods, DeepLinc, GCNLA, and OrgaCCC (Table 1).

**TABLE 1:** Performance comparison of CauSTI and baseline methods on three real-world spatial transcriptomic datasets. AUROC, ACC, and AP are reported as mean ± standard deviation over 50 independent runs. The best performance for each metric is highlighted in bold.

Method	seqFISH+			MERSCOPE			Xenium		
​	AUROC	ACC	AP	AUROC	ACC	AP	AUROC	ACC	AP
DeepLinc	69.12 ± 1.08	70.43 ± 1.04	63.34 ± 1.31	80.08 ± 1.91	78.23 ± 1.24	74.16 ± 1.35	82.06 ± 1.33	79.64 ± 2.42	75.93 ± 1.61
GCNLA	84.06 ± 1.07	81.83 ± 1.44	78.72 ± 1.53	86.04 ± 0.84	83.78 ± 1.75	81.43 ± 1.52	86.97 ± 0.92	84.58 ± 0.86	82.13 ± 2.05
OrgaCCC	84.96 ± 0.85	82.47 ± 1.13	79.83 ± 2.02	88.07 ± 1.17	85.63 ± 0.78	83.38 ± 1.28	87.04 ± 0.83	84.87 ± 1.22	82.64 ± 1.71
CauSTI	**86.27** ± **1.03**	**84.18** ± **0.65**	**82.43** ± **1.10**	**91.14** ± **0.65**	**88.73** ± **1.09**	**87.62** ± **2.03**	**92.08** ± **0.63**	**89.46** ± **0.78**	**88.37** ± **0.85**

Across all three datasets, CauSTI achieved the best overall performance. On the seqFISH + dataset, CauSTI obtained an AUROC of 86.27%, an ACC of 84.18%, and an AP of 82.43%, outperforming the strongest baseline, OrgaCCC, which achieved 84.96% AUROC, 82.47% ACC, and 79.83% AP. This indicates that the proposed spatial-causal modeling strategy improves both ranking performance and binary interaction prediction in spatially resolved cell-cell communication inference.

On the MERSCOPE dataset, CauSTI further improved the performance, reaching 91.14% AUROC, 88.73% ACC and 87.62% AP. Compared with OrgaCCC, the best-performing baseline on this dataset, CauSTI improved AUROC by 3.07 percentage points, ACC by 3.10 percentage points, and AP by 4.24 percentage points. The larger gain in AP suggests that CauSTI is particularly effective in prioritizing true interacting cell pairs under class-imbalanced conditions.

Consistent improvements were also observed on the Xenium dataset. CauSTI achieved the highest AUROC, ACC, and AP values, with 92.08%, 89.46%, and 88.37%, respectively. In comparison, OrgaCCC and GCNLA showed similar AUROC values around 87% , while DeepLinc obtained 82.06%. These results demonstrate that CauSTI maintains robust performance on high-resolution spatial transcriptomic data and generalizes well across different experimental platforms.

To further evaluate whether the observed performance improvements were statistically reliable, we conducted two-sided Welch’s t -tests comparing CauSTI with the strongest baseline method, OrgaCCC, across the 50 independent runs. Welch’s t -test was used because it does not assume equal variance between methods. Since nine dataset-metric comparisons were performed, Bonferroni correction was applied to control for multiple testing. As shown in Table 2, CauSTI achieved statistically significant improvements over OrgaCCC across all three datasets and all three evaluation metrics. All adjusted p values remained below 0.001, indicating that the performance gains were consistent across independent runs. These results further support the robustness of CauSTI compared with the strongest competing baseline.

**TABLE 2:** Two-sided Welch’s t -test comparing CauSTI with the strongest baseline method, OrgaCCC, across three datasets and three evaluation metrics. Each comparison was computed using the reported mean and standard deviation over 50 independent runs.

Dataset	Metric	CauSTI	OrgaCCC	t	df	p
seqFISH+	AUROC	86.27±1.03	84.96±0.85	6.94	94.6	<0.001
seqFISH+	ACC	84.18±0.65	82.47±1.13	9.28	78.2	<0.001
seqFISH+	AP	82.43±1.10	79.83±2.02	7.99	75.7	<0.001
MERSCOPE	AUROC	91.14±0.65	88.07±1.17	16.22	76.6	<0.001
MERSCOPE	ACC	88.73±1.09	85.63±0.78	16.35	88.8	<0.001
MERSCOPE	AP	87.62±2.03	83.38±1.28	12.49	82.6	<0.001
Xenium	AUROC	92.08±0.63	87.04±0.83	34.20	91.4	<0.001
Xenium	ACC	89.46±0.78	84.87±1.22	22.41	83.3	<0.001
Xenium	AP	88.37±0.85	82.64±1.71	21.22	71.8	<0.001

Overall, CauSTI consistently outperformed all competing methods across the three datasets and three evaluation metrics. The improvement over DeepLinc was especially pronounced, suggesting that replacing a conventional spatial graph autoencoding strategy with a spatial-causal prior-guided framework provides a more effective representation for cell-cell interaction prediction. The stable standard deviations across repeated runs also indicate that the proposed model is robust to random initialization and training variability.

### 4.6 Ablation study

To investigate the contribution of the causal prior and the directional regularization term, we conducted ablation experiments on three real-world spatial transcriptomic datasets, including seqFISH+, MERSCOPE, and Xenium. Three model variants were compared: the full CauSTI model, CauSTI without the causal prior (WCP), and CauSTI without the direction loss (WDL). WCP removes the ligand-receptor-derived causal-prior information from the model, while WDL removes the directional regularization term that suppresses unsupported bidirectional predictions.

As shown in Figure 2, the full CauSTI model consistently achieved the best performance across all datasets and metrics. On seqFISH+, CauSTI showed higher median AUROC, ACC, and AP than both WCP and WDL, indicating that the integration of causal-prior information and direction-aware optimization improves spatial cell-cell interaction prediction. The performance drop of WCP was more evident for AP, suggesting that ligand-receptor-derived causal priors are particularly useful for ranking high-confidence interacting cell pairs under class-imbalanced conditions.

**FIGURE 2:**
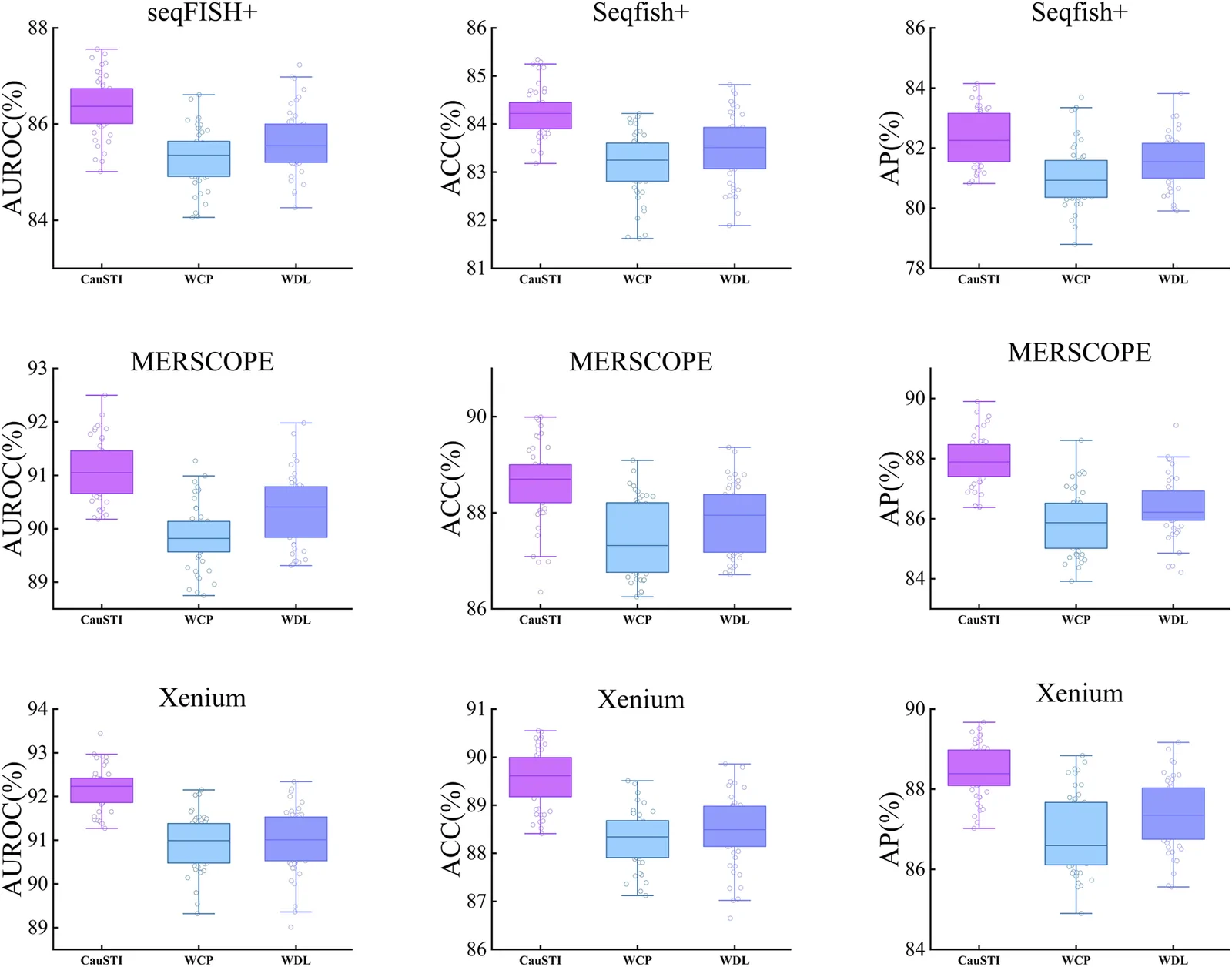
Ablation study of CauSTI on seqFISH+, MERSCOPE, and Xenium datasets. Boxplots show the distributions of AUROC, ACC, and AP over 50 independent runs. WCP denotes the variant without the ligand-receptor-derived causal prior, and WDL denotes the variant without the direction loss.

Similar trends were observed on the MERSCOPE dataset. CauSTI achieved the highest AUROC, ACC, and AP distributions, while WCP exhibited the largest reduction among the ablated variants. This result indicates that removing causal-prior information weakens the biological guidance available to the model and reduces its ability to prioritize reliable cell-cell communication signals. WDL also showed lower performance than the full model, demonstrating that the direction loss contributes to more stable and discriminative interaction inference.

On the Xenium dataset, CauSTI again outperformed both ablated variants across all three metrics. The gap between CauSTI and WCP confirms the importance of causal-prior integration, whereas the decline observed in WDL supports the role of directional regularization in refining directed interaction scores. Overall, these results demonstrate that both the causal prior and the direction loss are beneficial to the proposed framework. The consistent superiority of CauSTI over WCP and WDL across datasets suggests that the two components act complementarily: the causal prior provides biologically informed directional constraints, while the direction loss reduces ambiguous bidirectional predictions and improves the robustness of the inferred interaction network.

## 5 Conclusion

In this study, we proposed CauSTI, a causal spatial transcriptomic interaction inference framework for modeling direction-specific cell-cell communication from spatial transcriptomic data. Unlike conventional approaches that mainly rely on spatial proximity or ligand-receptor co-expression scores, CauSTI integrates spatial neighborhood information and ligand-receptor-derived causal priors into an end-to-end variational graph autoencoder framework. By combining spatial graph construction, causal-prior-guided representation learning, asymmetric link decoding, and directional regularization, CauSTI is able to infer ordered sender-receiver interaction probabilities between cells.

Extensive experiments on seqFISH+, MERSCOPE, and Xenium datasets demonstrated that CauSTI consistently achieved superior performance compared with DeepLinc, GCNLA, and OrgaCCC across AUROC, ACC, and AP. The improvement was particularly evident on high-resolution spatial transcriptomic datasets, indicating that the proposed spatial-causal modeling strategy can effectively capture both local tissue organization and biologically meaningful communication signals. In addition, ablation studies showed that removing either the causal prior or the direction loss led to performance degradation, confirming that ligand-receptor-derived causal constraints and direction-aware optimization are both important components of the framework.

Overall, CauSTI provides a flexible and interpretable computational framework for reconstructing spatially constrained and direction-specific cell-cell interaction networks. The inferred interaction landscape can help characterize tissue-level communication patterns and provide candidate signaling relationships for downstream biological investigation. A current limitation is that the main benchmark uses spatial adjacency as a uniform proxy for candidate interactions, which enables fair comparison across methods but cannot fully establish genuine signaling activity. Future work will focus on incorporating cell-type-specific regulatory programs, extending the model to multi-sample and temporal spatial transcriptomic data, and validating predicted interactions using independent ligand-target, perturbation, or imaging-based experimental evidence.

## Data Availability

The original contributions presented in the study are included in the article/supplementary material, further inquiries can be directed to the corresponding author.
